# Investigating the Molecular Basis of Siah1 and Siah2 E3 Ubiquitin Ligase Substrate Specificity

**DOI:** 10.1371/journal.pone.0106547

**Published:** 2014-09-09

**Authors:** Anupriya Gopalsamy, Thilo Hagen, Kunchithapadam Swaminathan

**Affiliations:** 1 Department of Obstetrics and Gynecology, Yong Loo Lin School of Medicine, National University of Singapore, Singapore, Singapore; 2 Department of Biochemistry, Yong Loo Lin School of Medicine, National University of Singapore, Singapore, Singapore; 3 Department of Biological Sciences, National University of Singapore, Singapore, Singapore; Georgia Regents University, United States of America

## Abstract

The Siah1 and Siah2 E3 ubiquitin ligases play an important role in diverse signaling pathways and have been shown to be deregulated in cancer. The human Siah1 and Siah2 isoforms share high sequence similarity but possess contrary roles in cancer, with Siah1 more often acting as a tumor suppressor while Siah2 functions as a proto-oncogene. The different function of Siah1 and Siah2 in cancer is likely due to the ubiquitination of distinct substrates. Hence, we decided to investigate the molecular basis of the substrate specificity, utilizing the well-characterized Siah2 substrate PHD3. Using chimeric and mutational approaches, we identified critical residues in Siah2 that promote substrate specificity. Thus, we have found that four residues in the N-terminal region of the Siah2 substrate binding domain (SBD) (Ser132, His150, Pro155, Tyr163) are critical for substrate specificity. In the C-terminal region of the SBD, a single residue, Leu250, was identified to promote the specific binding of Siah2 SBD to PHD3. Our study may help to overcome the challenges in the identification of Siah2 specific inhibitors.

## Introduction

SIAH (Seven in Absentia Homolog) is a mammalian homolog of Seven in Absentia (SINA), a *Drosophila* protein that has a function in eye development [1]. Two SINA homologs have been identified in the human genome, Siah1 and Siah2 [2], both of which encode functional proteins. The Siah family of proteins are evolutionarily conserved E3 ubiquitin ligases that have recently been implicated in various cancers and show promise as anticancer drug targets.

The Siah family proteins contain an N-terminal RING domain followed by two Zinc fingers and a C-terminal substrate binding domain (SBD) [3]. The crystal structure of the Siah1 SBD has been determined [4]–[7] and contains a fold that has not been observed in other E3 structures [8], [9]. To date the structure of Siah2 has not been determined. However, these two proteins share high sequence similarity and presumably high structural homology. The high level of sequence conservation between Siah1 and Siah2 is reflected in similar functional roles by sharing a number of ubiquitination substrates [10], [11]. However, both Siah1 and Siah2 also have specific substrates. Moreover, the expression of Siah1 and Siah2 is differentially regulated, providing further support for different functional roles. For instance, Siah1 is induced by p53 upon genomic stress due to DNA damage, while Siah2 is induced by hypoxia, estrogens, etc. [12]–[14]. One of the recent studies reports that estrogen increases the protein and mRNA expression of Siah2 but not of Siah1 [15]. A report investigating the physiological function of Siah1 and Siah2 by generating knock-out mice demonstrated that deletion of Siah1 results in sub-viability and growth retardation. In contrast, Siah2 knock-out mice are completely viable. Of note, Siah2 Siah1 double knock-out mice die at birth [16]. This supports the notion that Siah1 and Siah2 proteins have both distinct and overlapping functions.

Siah1 and Siah2 are known to function as E3 ubiquitiin ligases that mediate the ubiquitination of diverse cellular substrates. In mammals, more than 30 substrates of the Siah ubiquitin ligases have been identified [17]–[19]. For instance, the Siah proteins regulate the ubiquitination-dependent degradation of transcriptional repressors such as NcoR/TIEG-1, transcriptional activators, for instance β-catenin, the netrin receptor, the microtuble-associated motor protein Kid as well as multiple other proteins. By controlling the stability of these sustrate proteins, Siah1 and Siah2 regulate an array of cellular functions, such as angiogenesis, DNA damage response, mitochondrial dynamics and Ras and estrogen-receptor (ER) dependent signaling.

The role of the Siah1 E3 ubiquitin ligase in cancer is currently poorly understood. However, Siah1 is more often described as a tumour suppressor [20]. For instance, the expression levels of Siah1 have been reported to be downregulated in various cancers. Also, inhibition or low levels of Siah1 have been shown to negatively regulate apoptosis, thereby promoting cancer progression [21]–[24]. In contrast to the role of Siah1, Siah2 has been described to function as a proto-oncogene. Growing evidence highlights the functional role of Siah2 in promoting the progression of multiple types of cancer, including breast [25]–[27], lung [28], pancreatic [29], prostate [30], [31], liver [32] cancer and melanoma [13].

The different roles of Siah1 and Siah2 in cancer are likely mediated through the ubiquitination of distinct substrates. For instance, Siah1 but not Siah2, polyubiquitinates and degrades ELL2 [33]. Siah1 and Siah2 SBD are highly conserved with 86% sequence similarity and the molecular basis for the specificity in substrate recognition by Siah1 and Siah2 is currently unknown. One of the Siah2 specific substrates is prolyl hydroxylase 3 (PHD3). PHD3 belongs to a family of oxygen and 2-oxoglutarate dependent prolyl hydroxylases, which also includes PHD1 and PHD2 [34]. These prolyl hydroxylases have been shown to function as cellular oxygen sensors by hydroxylating a number of substrates, including Hypoxia Inducible Factor 1α (HIF-1α). Hydroxylation of HIF-1α at two conserved proline residues leads to its rapid degradation. It has been shown that the E3 ligase Siah2 preferentially ubiquitinates PHD3 under hypoxic conditions, thus leading to PHD3 degradation and consequently to HIF-1α stabilization. Thus Siah2 plays an important role in hypoxia dependent signaling, and this is likely to contribute to its tumor promoting activity [35], [36].

Given the different roles of Siah1 and Siah2 in cancer and their different cellular functions, it is important to understand the structural basis of their substrate specificity and to design Siah2 specific inhibitors. Hence, in this study we decided to investigate the molecular basis underlying the substrate specificity of Siah2 in comparison with Siah1 using the well characterised substrate PHD3.

## Materials and Methods

### Plasmid constructs

The pcDNA3.1 FLAG-SBD of human Siah2 (residues 130–392) and full-length (1–394) were constructed by PCR amplification of Siah2 cDNA fragments separately from the pCMV-SPORT6 plasmid (Thermo Scientific OpenBiosystems) with a Hind III-containing forward primer and an XbaI containing reverse primer. The HindIII-Siah2-XbaI fragments were then subcloned into the FLAG-pcDNA3.1 plasmid. Similarly, FLAG-SBD of Siah1 (90–292) and full-length (1–292) were constructed using the same restriction sites. The HA-PHD3 plasmid, carrying a C-terminal HA tag, was constructed by PCR amplification of human PHD3 from HEK293 cell cDNA, including a KpnI site and an XbaI site plus HA tag sequence in the 5′ and 3′ primers, respectively. The PCR product was inserted into the KpnI and XbaI sites of pcDNA3.

### Cell culture and transfection

Human embryonic kidney 293T (HEK293T) were grown at 37°C and 5% CO_2_ in Dulbecco's modified Eagle's medium (Invitrogen), supplemented with 10% fetal bovine serum (FBS) (HyClone), L-glutamine (Invitrogen) and penicillin/streptomycin (Invitrogen).

DNA plasmids were transiently co-transfected in subconfluent HEK293T cells plated in a 60 mm plate with the GeneJuice transfection reagent (Novagen) according to the manufacturer's instructions. Empty pcDNA3.1 vector was also co-transfected as a control. Cells were lysed 48 hours after transfection.

### Co-immunoprecipitaction

Cells were washed with cold PBS and lysed 2 days post-transfection with lysis buffer containing 25 mM Tris-HCL (pH 7.5), 3 mM EDTA, 2.5 mM EGTA, 20 mM NaF, 1 mM Na_3_VO_4_, 20 mM sodium β-glycerophosphate, 10 mM sodium pyrophosphate, 0.5% Triton X-100, 0.1% β-mercaptoethanol and Roche protease inhibitor cocktail. Lysates from transfected cells were pre-cleared by centrifugation and were added to anti-FLAG or anti-HA M2 monoclonal antibody coupled agarose beads to immunoprecipitate the FLAG-Siah2 or HA-PHD3. Samples were tumbled at 4°C for 1 hour and washed four times with NP40 lysis buffer containing 20 mM Tris (pH 7.5), 50 mM NaCl, 0.5 mM EDTA, 5% glycerol, 0.5% NP40 and once with buffer containing 50 mM Tris (pH 7.5).

### GST-SBD expression

To prepare the recombinant GST-Siah2 SBD (residues 130–322) protein, a bacterial expression plasmid construct of GST-Siah2 was generated in the pGEX-6P-1 vector. This construct was transformed into *E. coli* BL21 and induced with 0.2 mM IPTG at 18°C overnight. Bacterial pellets were harvested, sonicated and lysed in 50 mM Tris-HCl (pH 8.0), 100 mM NaCl, 2 mM dithiothreitol containing a protease inhibitor cocktail (Sigma).

### GST pull down

For GST pull down assay, GST-Siah2 SBD was allowed to bind to glutathione sepharose beads (GSH) (GE Healthcare) for 30 min at 4°C in binding buffer containing 50 mM Tris-HCl (pH 8), 150 mM NaCl, 1 mM DTT, 5% glycerol, 0.1% Triton X-100. Cell lysate from HEK293T cells transfected with HA-PHD3 was incubated with the GST-Siah2 fusion proteins, immobilized on glutathione sepharose beads, for 1 hour at 4°C. GST alone was used as a control. After binding, the resin was washed three times in binding buffer, and then heated in Laemmli sample buffer for 5 min at 95°C. Samples were separately resolved in 12% PAGE and western blotted using an anti-HA antibody.

### Domain swapping using fusion PCR and mutagenesis

A three step fusion PCR [37] procedure was employed to create the fusion proteins, SBD[S1]^NT^[S2]^CT^ (SBD with Siah1 N-terminus and Siah2 C-terminus) and SBD[S2]^NT^[S1]^CT^ (SBD with the Siah2 N-terminus and Siah1 C-terminus) from the wild type pcDNA Siah1 and Siah2 constructs. Specific mutations were generated by site directed mutagenesis. Selected mutant SBD[S1]^NT^[S2]^CT^ and SBD[S2]^NT^[S1]^CT^ constructs were custom synthesized (Shanghai Shine Gene Molecular Biotech and Genscript).

### Homology modeling and docking of Siah2 SBD and PHD3

The three dimensional (3D) models of Siah2 SBD and PHD3 were prepared by homology modeling using the SWISS-MODEL automated protein modeling server (http://swissmodel.expasy.org/) [38]. The model of the complex of Siah2 SBD/PHD3 was constructed using the ClusPro program [39], which is composed of three steps: docking using a Fast Fourier Transform-based algorithm; energy filtering using a combination of desolvation and electrostatic energies; clustering steps to discriminate against false positives and reduce the set of configurations to near-native structures. The models with a balanced scoring function were accepted and the top ranked model was analyzed for interacting residues using Pymol [40] and Pdbsum [41].

## Results

### SBD of Siah2 alone can independently interact with PHD3

**Figure 1:**
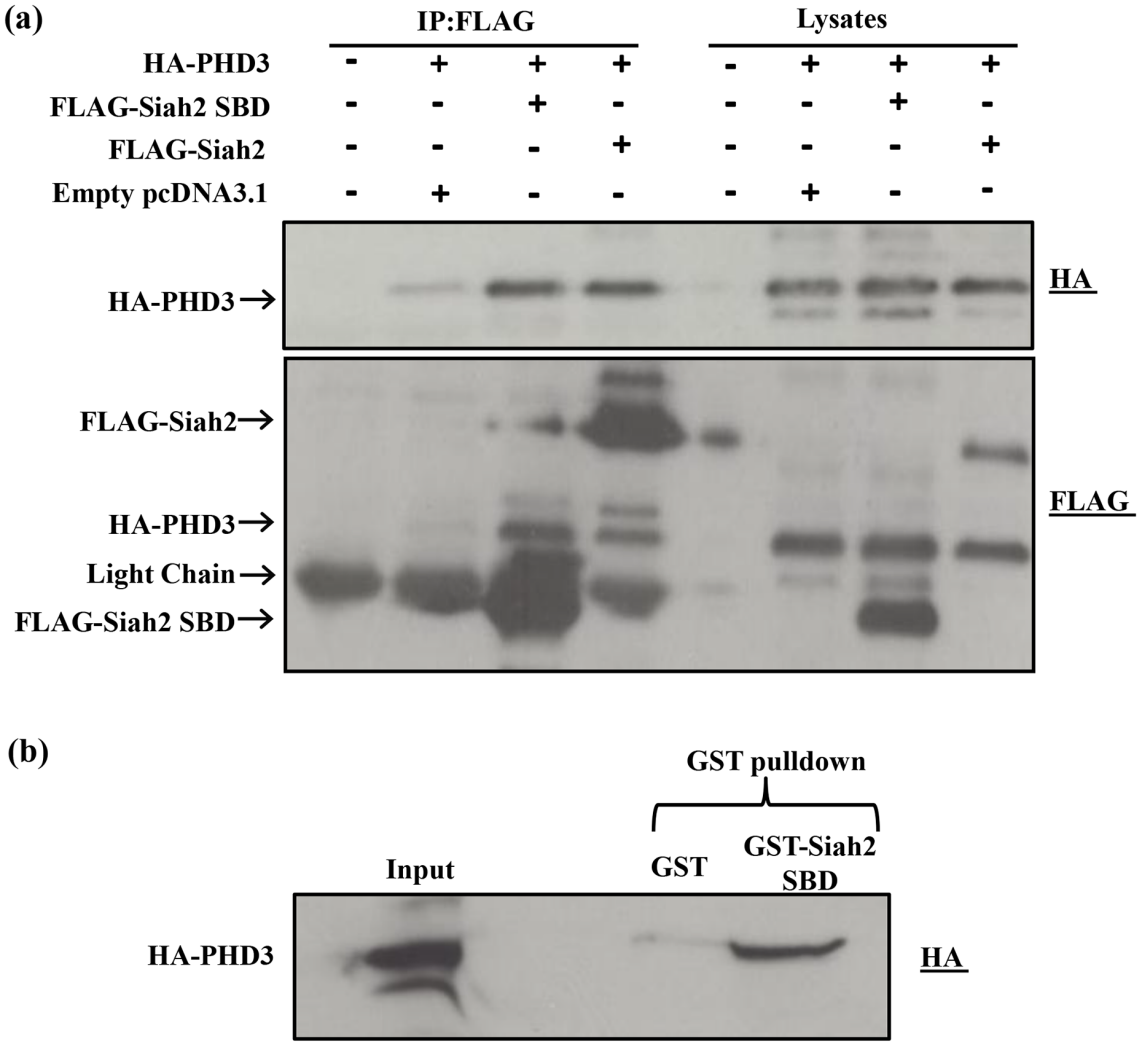
Interaction of Siah2 with PHD3. (**a**) HEK293 cells were transfected in 60-mm cell culture plates for 2 days with the indicated expression plasmids. The cells were lysed, and the lysates were subjected to FLAG immunoprecipitation (IP), as described under “Materials and Methods”. Aliquots of the cell lysates and immunoprecipitates were analyzed by western blotting with the anti-HA antibody. Both full length Siah2 and Siah2 SBD bind to PHD3 to the same extent. In the IP, the presence of the faint band in the empty vector lane is due to non-specific binding of PHD3. The same membrane was reblotted with FLAG antibody to detect FLAG tagged Siah2 proteins. (**b**) GST-Siah2 SBD pulldown of HA-PHD3. Cell lysate of HEK293 cells transfected with HA-PHD3 was incubated with GST-Siah2 SBD immobilized on GSH agarose beads and the reaction was performed as described under “Material and Methods”. The empty expression vector alone was expressed as a GST control for non-specific binding of HA PHD3. After the incubation, the lysate was removed, the GSH-agarose beads were washed, and bound HA-PHD3 was analyzed by Western blotting using anti HA antibody. The pull down assay confirmed the interaction of Siah2 SBD with PHD3.

Given that PHD3 is a well characterized substrate of Siah2, we chose this substrate for our studies. We used co-immunoprecipitation to analyze the interaction between Siah2 and PHD3. Two Siah2 plasmid constructs, full length and SBD with an N-terminal FLAG tag, were generated. Subsequently, cells were cotransfected with the constructs encoding for full length FLAG-Siah2 or FLAG- Siah2 SBD and HA-PHD3. Anti-FLAG M2 agarose beads were used to immunoprecipiate the Siah2-PHD3 protein complex. The complex was analyzed using western blots with HA antibody to detect PHD3 bound to Siah2. When comparing the ratio of PHD3 in the FLAG-immunopreciptates to that in the lysate, an enrichment of PHD3 protein was seen in the immunoprecipitates, suggesting strong binding of Siah2 to PHD3 (Fig. 1a). Furthermore, it was found that the amounts of PHD3 bound to full length Siah2 and the SBD of Siah2 were similar. The comparable binding of PHD3 to full length and SBD of Siah2 suggests that the substrate binding domain alone is sufficient for the interaction with PHD3. Hence, in further experiments we focused on the interaction between Siah2 SBD and PHD3.

To confirm the interaction between Siah2 SBD and PHD3, we also carried out GST pull down assay, using recombinant GST tagged SBD of Siah2 and lysates from cells transfected with PHD3. As shown in Fig. 1b, specific *in vitro* binding of PHD3 to Siah2 SBD was detected.

### Siah1-PHD3 interaction

**Figure 2:**
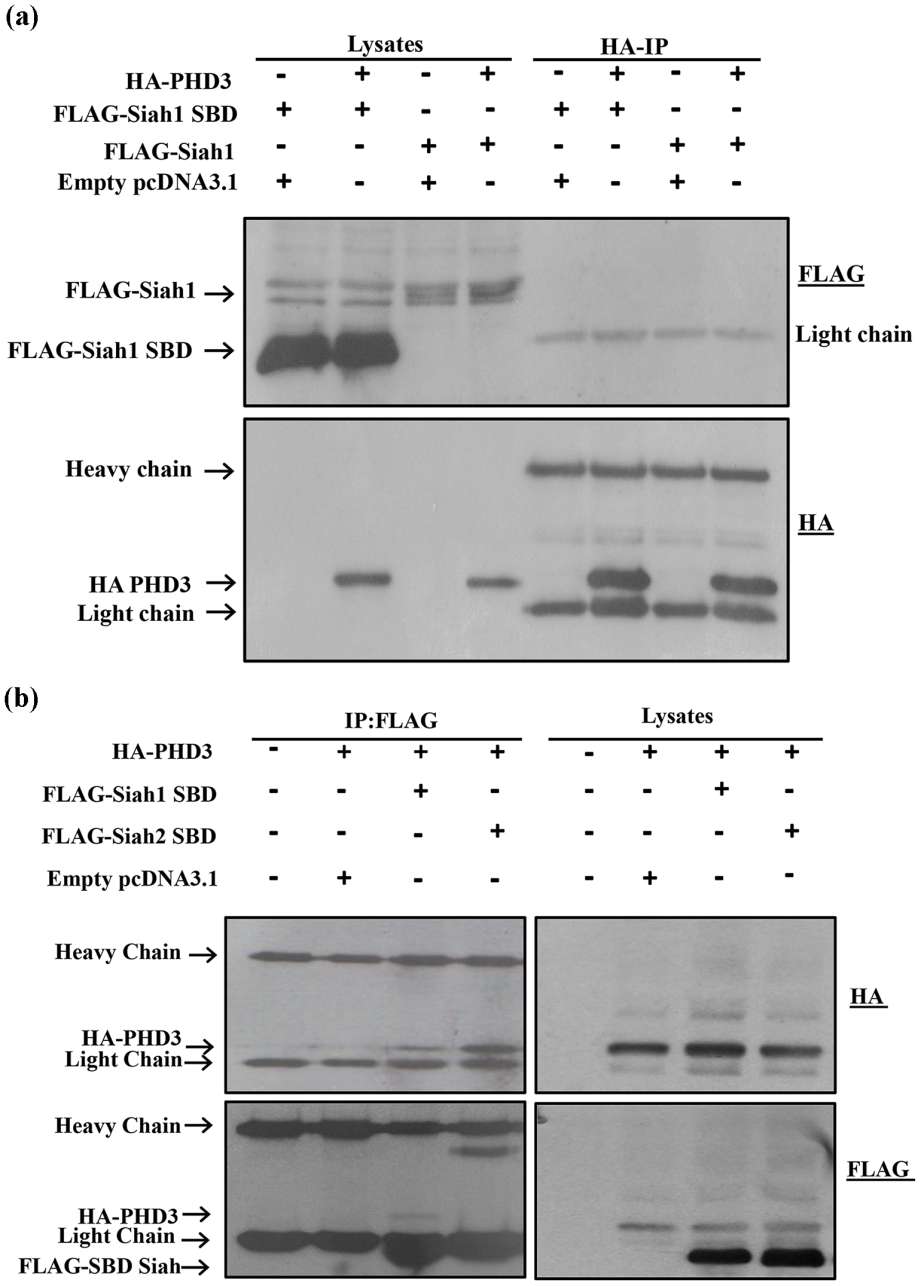
Siah1 exhibits weak binding compared to Siah2 with PHD3. HEK293T cells were transfected in 60-mm cell culture plates for 2 days with expression plasmids for the proteins indicated at the top of each panel. (**a**) Cell lysates were subjected to HA-IP and aliquots of the cell lysates and immunoprecipitates were analyzed by western blotting with the anti-FLAG antibody. Both the Full length and Siah1 SBD did not show binding to PHD3 (**b**) The lysates were subjected to reciprocal FLAG-IP. Immunoprecipitates and aliquots of the cell lysates were analyzed by Western blotting with anti-HA and anti-FLAG antibodies. In the IP, FLAG-SBD overlaps with the IgG light chain. Compared to Siah2 SBD, only weak binding of Siah1 SBD to PHD3 was observed.

It has been reported that Siah2 is more efficient than Siah1 in inducing the degradation of PHD3 [27]. However, no direct interaction of Siah1 with PHD3 has been reported so far. Hence, we performed co-immunoprecipitation assay to check the binding of both full length and the SBD of Siah1 with PHD3. To this end, HEK293T cells were cotransfected with the corresponding expression constructs, followed by HA immunoprecipitation. However, no binding between PHD3 and Siah1 was detected (Fig 2a). Subsequently, reciprocal co-immunoprecipitation was performed to compare the interaction of Siah1 and Siah2 with PHD3. Thus, HEK293T cells were cotransfected with the FLAG-Siah1 SBD and FLAG-Siah2 SBD and HA-PHD3 expression constructs, followed by FLAG immunoprecipitation. Interestingly we observed only a weak interaction of Siah1 SBD with PHD3 when compared to the binding of Siah2 SBD to PHD3 (Fig. 2b) Hence, the data obtained by immunoprecipitation assays suggest that there is a marked difference in the binding affinities of Siah1 and Siah2 SBD for PHD3.

### Interaction of chimeric forms of Siah1 and Siah2 SBD with PHD3

**Figure 3:**
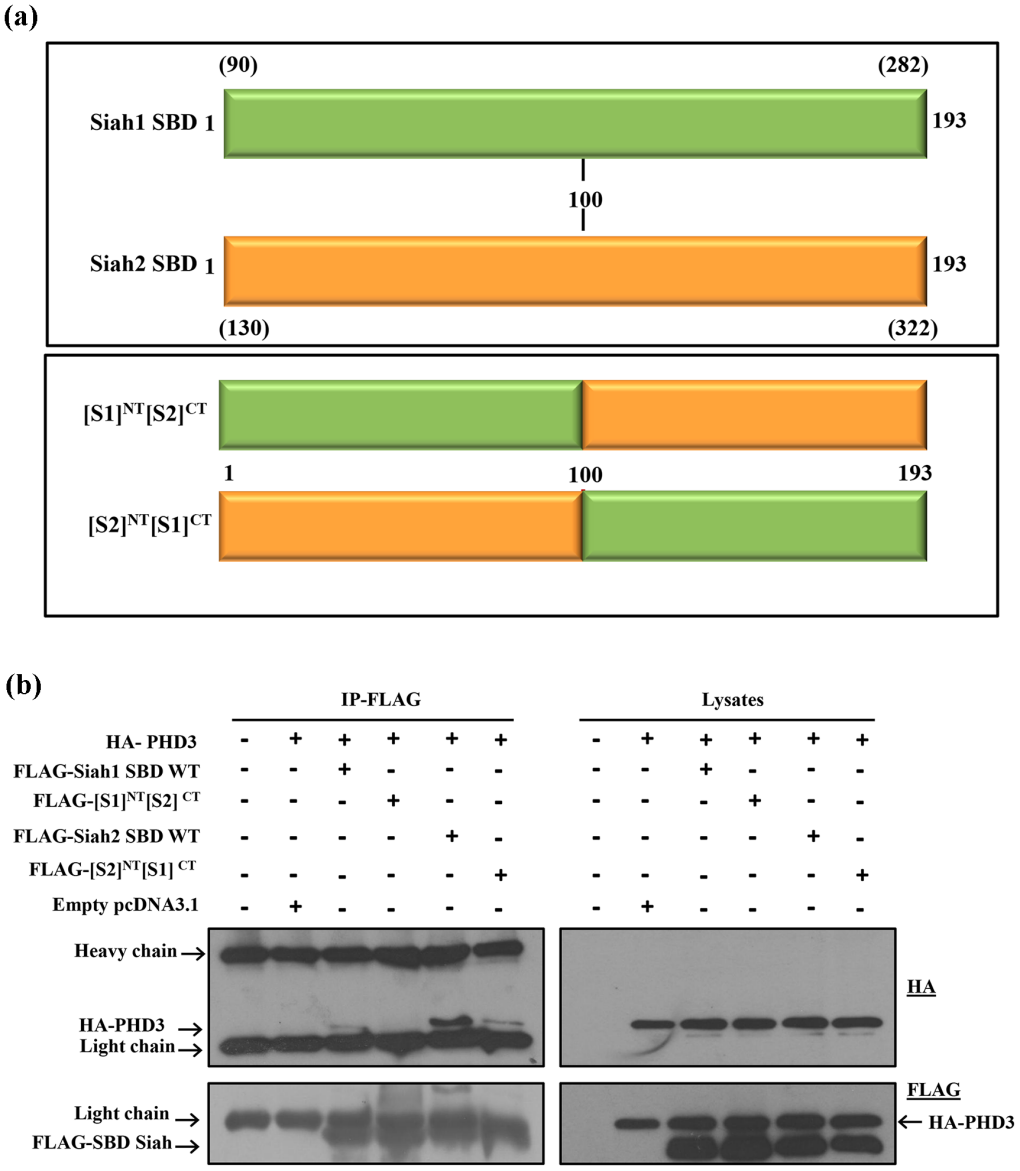
Interaction of wild type (WT) and chimeric Siah proteins with PHD3. (**a**) Diagrammatic representation of the WT Siah1 and Siah2 SBD, which comprises of 1–193 residues, and the chimeric forms of Siah1 and Siah2 SBD, SBD[S1]^NT^[S2]^CT^ and SBD[S2]^NT^[S1]^CT^. Corresponding original residue numbers are given in parentheses. (**b**) HEK293T cells were transfected in 60-mm cell culture plates for 2 days with the indicated expression plasmids. 48 hours after transfection, the cells were lysed and cell lysates were subjected to FLAG immunoprecipitation (IP). Immunopercipiates and the aliquotes of lysates were then immunoblotted using indicated antibodies. Both the chimeric forms lost binding to PHD3 as compared to wild type.

In order to determine which regions of the SBD are critical for the interaction with PHD3, we generated chimeric forms of Siah1 and Siah2 SBD. The SBD of Siah1 and Siah2 comprises of residues 90-282 (193 aa) and 130-322 (193 aa), respectively. To avoid confusion, residue numbers for both the Siah1 and Siah2 SBD are labeled and hereafter referred as 1-193, unless stated otherwise. Residues 101–193 of Siah1 were swapped with the corresponding region of Siah2 and vice versa to obtain the Siah1 N-terminus/Siah2 C-terminus SBD chimera ([S1]^NT^[S2]^CT^) and the Siah2 N-terminus/Siah1 C-terminus SBD chimera ([S2]^NT^[S1]^CT^) constructs, respectively (Fig. 3a). We used co-immunoprecipitation to investigate the interactions of these two chimeras with PHD3 (Fig 3b). Consistent with our previous results, the wild type Siah2 SBD interacted with PHD3 strongly compared to the weak binding of Siah1 SBD. The binding of both chimeric forms [S1]^NT^[S2]^CT^ and [S2]^NT^[S1]^CT^ with PHD3 was markedly reduced. Thus, the chimera that lacks the N- or C-terminal region of Siah2 SBD lost its binding with PHD3 compared to wild type Siah2. These results suggest that both regions of SBD of Siah2, 1–100 and 101–193, are important for binding with PHD3.

### Identification of critical residues in the Siah2 SBD that mediate substrate specificity

**Figure 4:**
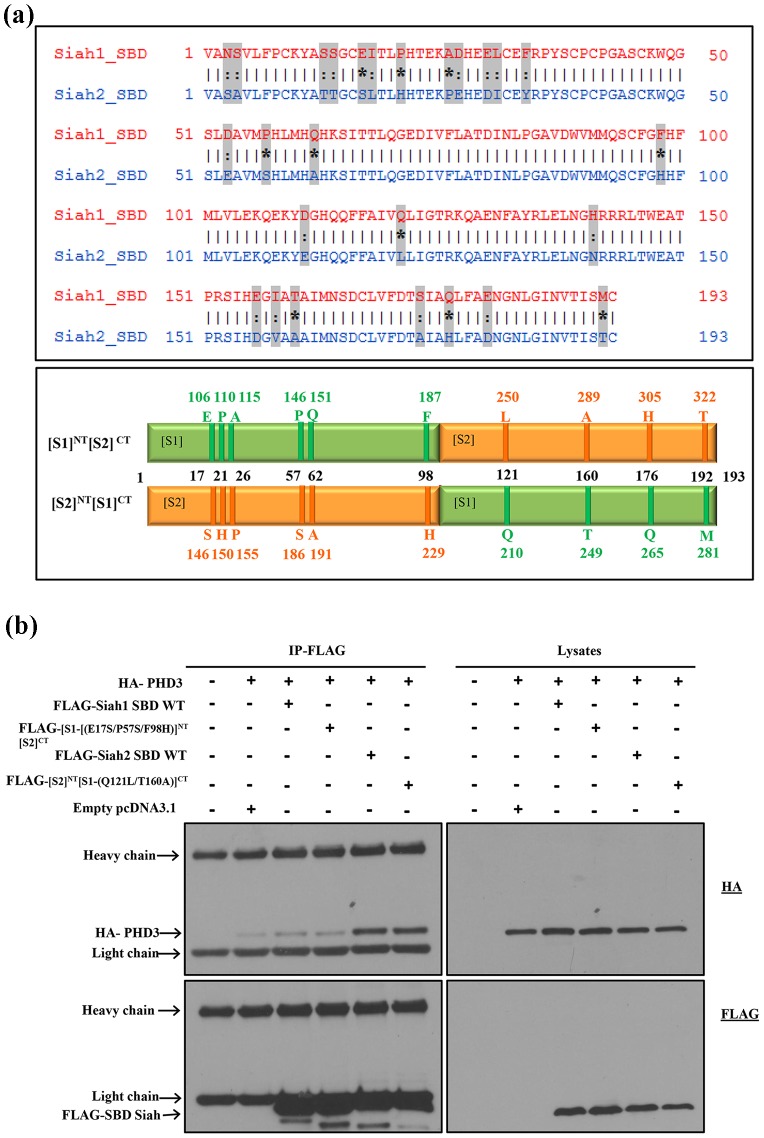
Effect of mutations in Siah1 and Siah2 SBD Chimeras on binding with PHD3. (**a**) Pairwise sequence alignment of Siah1 and Siah2 SBD was performed by EMBOSS Needle tool. The 26 amino acids that are unique in Siah1 and Siah2 SBD are highlighted in grey. Dissimilar amino acids are highlighted by ‘*’. Similar amino acids are highlighted by ‘:’ and identical amino acids are highlighted by ‘|’ (top panel). The 10 dissimilar amino acids between Siah1 and Siah2 SBD are shown in diagrammatic representation of the chimeric forms, SBD[S1]^NT^[S2]^CT^ and SBD[S1]^NT^[S2]^CT^(bottom panel). The original residue numbers are labeled in the respective colors (**b**) HEK293T cells were transfected with the indicated expression plasmids, followed by FLAG immunoprecipitation (IP) of cell lysates. Immunoprecipitates and lysates were then analyzed by western blotting using the indicated antibodies. The N-terminal mutant chimera, [S1-(E17S/P57S/F98H)]^NT^[S2]^CT^ did not regain binding to PHD3 and the C-terminal mutant chimera, [S2]^NT^[S1-(Q121L/T160A]^CT^ regained complete binding to PHD3 equivalent to WT Siah2 SBD.

Next, we performed mutation studies with the chimeric forms to investigate the molecular basis of the substrate specificity. Hence, to identify the critical residues in the Siah2 SBD, pairwise sequence alignment of Siah1 and Siah2 SBD was performed by the EMBOSS Needle tool [42]. Based on the alignment, 26 amino acids were found to be different, of which 10 residues are dissimilar and 16 are similar (Fig. 4a top panel). Therefore, we first focused on the 10 dissimilar amino acids in our mutation studies.

Out of the 10 dissimilar residues, 6 residues are in the N-terminal region and 4 residues are in the C-terminal region of the SBD (Fig. 4a bottom panel). Mutations were generated in a stepwise manner to identify the critical residues that might confer substrate specificity. First, three of the 6 N-terminal dissimilar amino acids of Siah1 in [S1]^NT^[S2]^CT^ chimera (Glu17, Pro57, Phe98) were mutated back to the corresponding Siah2 residues, giving rise to the chimera [S1-(E17S/P57S/F98H)]NT[S2]CT. Similarly, out of the 4 dissimilar amino acids of Siah1 in the [S2]^NT^[S1]^CT^ chimera, two (Gln121, Thr160) were mutated back to the corresponding Siah2 residues, resulting in [S2]^NT^[S1-(Q121L/T160A]^CT^. Next, we carried out co-immunoprecipitation studies with the mutated chimeras to determine whether the introduced mutations would improve the binding to PHD3. The results show that [S1-(E17S/P57S/F98H)]^NT^[S2]^CT^ did not regain binding, suggesting other residues in the N-terminal SBD could be important. On the other hand, [S2]^NT^[S1-(Q121L/T160A]^CT^ regained binding with PHD3 to levels equivalent to the binding of wild type Siah2 SBD (Fig. 4b). This indicates the crucial role of the Siah2 residues Leu121 and Ala160 in binding to PHD3. Hence, further single point mutations of S2]^NT^[S1-(Q121L/T160A]^CT^ and selected mutations of [S1-(E17S/P57S/F98H)]NT[S2]CT were generated to identify the specific residues that play a critical role in selective binding.

**Figure 5:**
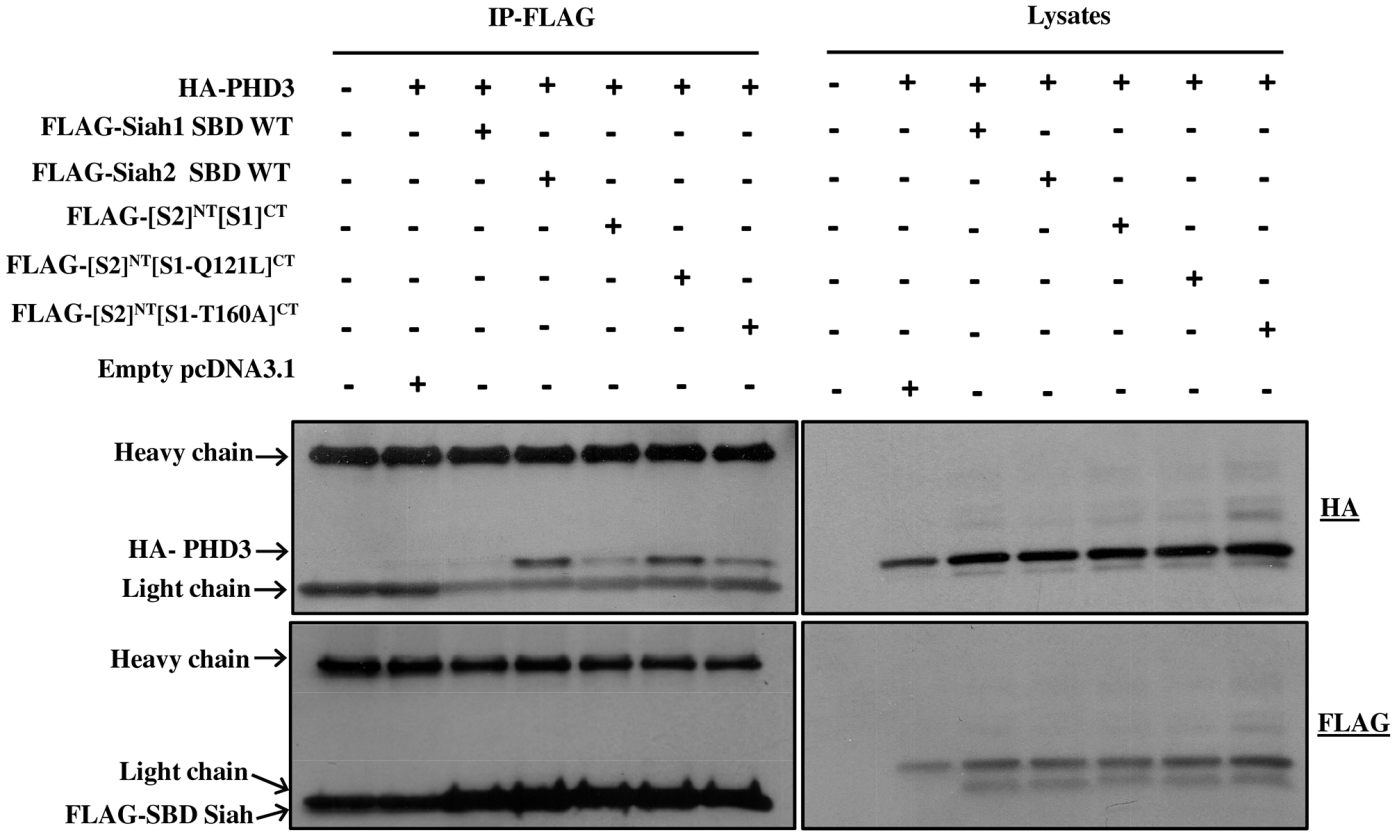
Effect of mutations in the C-terminal region of the SBD on binding with PHD3. HEK293T cells were transfected with the expression plasmids for the indicated proteins. The cells were lysed followed by FLAG immunoprecipitation (IP) of cell lysates. Immunoprecipitates and lysates were then analyzed by western blotting using the indicated antibodies. The [S2]^NT^[S1-Q121L]^CT^ chimera regained binding equivalent to Siah2 SBD wild type. In contrast, [S2]^NT^[S1-T160A]^CT^ showed only a small increase in PHD3 binding. FLAG-SBD Siah in the IP was masked by the IgG light chain.

We first focused on the C-terminal SBD. As shown above, mutation of the Siah1 residues Gln121 and Thr160 to the corresponding residues in Siah2, Leu121 and Ala160, respectively, restored the binding to PHD3 to Siah2 SBD wild type levels. Therefore, we next investigated which of the two mutants (either one or both) is critical for PHD3 binding. To this end, two individual point mutants were generated, [S2]^NT^[S1-Q121L]^CT^ and [S2]^NT^[S1-T160A]^CT^. Subsequently, the binding of the two mutants to PHD3 was analysed by co-immunoprecipiation. It was observed that the Q121L mutant restored binding to the level of wild type Siah2 SBD. In contrast, T160A showed only a small increase in PHD3 binding. These results indicate the critical importance of Leu121 in the C-terminal region of the Siah2 SBD (Fig. 5).

**Figure 6:**
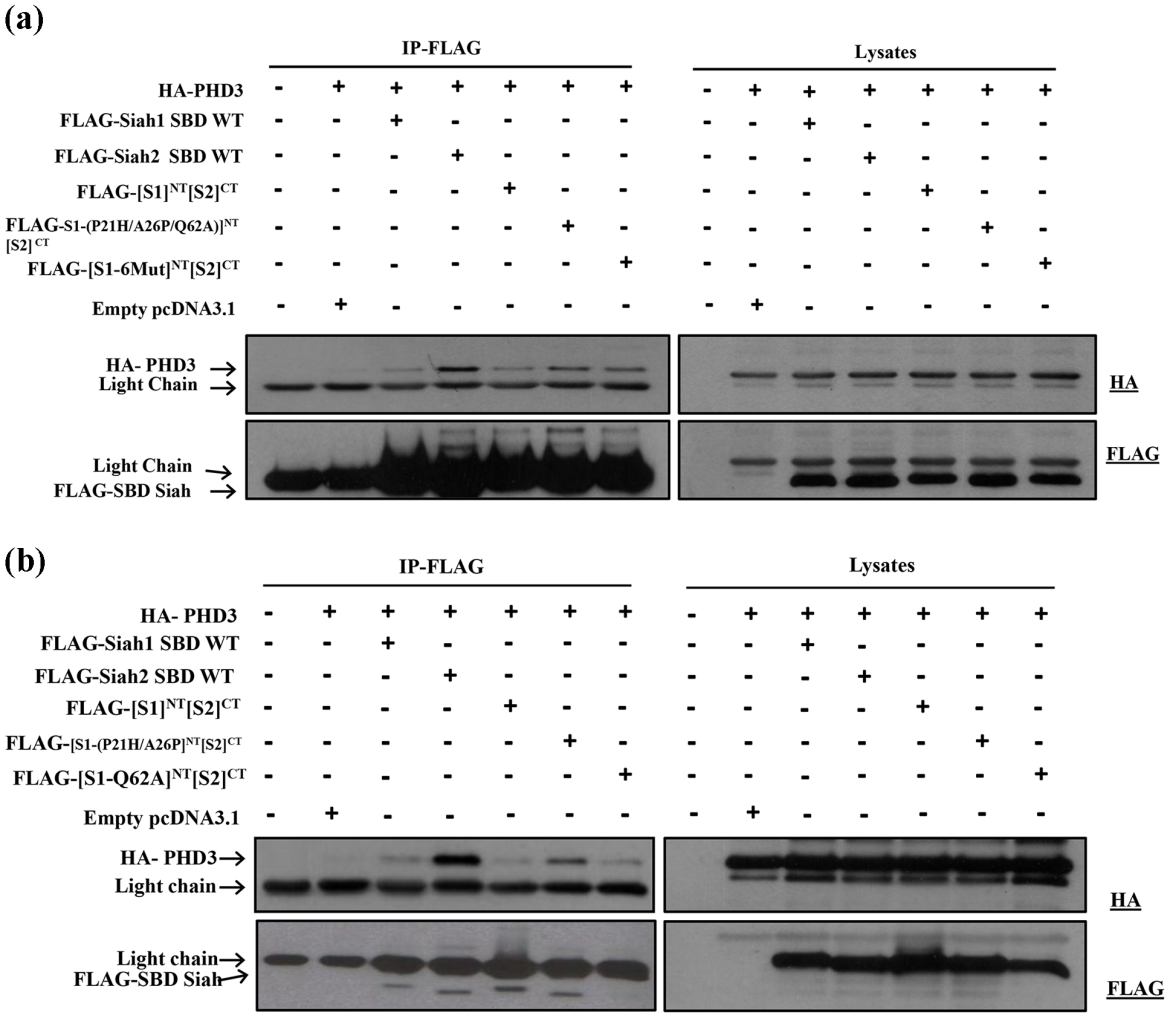
Effect of mutations in the N-terminal region of the SBD on binding with PHD3. HEK293T cells were transfected with the expression plasmids for the proteins indicated at the top of each panel. The cells were lysed and the cell lysates were subjected to FLAG-immunoprecipiation (IP). Immunoprecipitates and lysates were then analyzed by western blotting using the indicated antibodies. (**a**) Both [S1-(P21H/A26P/Q62A)]^NT^[S2]^CT^ and [S1-6Mut]^NT^[S2]^CT^ chimeras only partially regained binding to PHD3, compared to binding of wild type Siah2 SBD to PHD3. (**b**) Only the chimera with the P21H/A26P mutations regained partial binding with PHD3. In contrast, mutation of Q62A did not increase PHD3 binding. FLAG-SBD Siah in the IP was masked by the IgG light chain.

In subsequent experiments, we studied the involvement of critical residues in the N- terminal region of Siah2 SBD. Our initial chimera [S1-(E17S/P57S/F98H)]^NT^[S2]^CT^ did not exhibit any significant binding to PHD3. This indicates that other residues in the N-terminal SBD are important in conferring specificity for substrate binding to Siah2. We focused on the three remaining N-terminal dissimilar residues in the [S1]^NT^[S2]^CT^ chimera (Pro21, Ala26, Gln62). We generated a chimeric construct in which these three amino acids were mutated back to the corresponding residues in Siah2 SBD, [S1-(P21H/A26P/Q62A)]^NT^[S2]^CT^. In addition we also generated a chimera in which all 6 dissimilar residues were mutated back to those in Siah2, [S1-6Mut]^NT^[S2]^CT^ (where 6Mut corresponds to E17S/P21H/A26P/P57S/Q62A/F98H) (Fig. 4a). Subsequently, the interaction between these mutant constructs and PHD3 was investigated using co-immunoprecipitation. Both [S1-(P21H/A26P/Q62A)]^NT^[S2]^CT^ and [S1-6Mut]^NT^[S2]^CT^ chimeras only partially regained binding to PHD3, compared to binding of wild type Siah2 SBD to PHD3 (Fig. 6a). Furthermore, it was found that the chimeras with the three and six mutations showed similar binding to PHD3. This suggests that the partial regaining of the interaction with PHD3 is likely due to the mutant residues in [S1-(P21H/A26P/Q62A)]^NT^[S2]^CT^. These results suggest an involvement of His21, Pro26 and/or Ala62, whereas Ser17, Ser57 and His98 are not involved in the interaction of Siah2 with PHD3.

Subsequently we studied the importance of His21, Pro26 and Ala62 in the C-terminal region of Siah2 SBD. Thus, we generated two mutant constructs. In the first construct, Pro21 and Ala26 in the Siah1 SBD were mutated to the corresponding residues in Siah2, His21 and Pro26, respectively ([S1-(P21H/A26P)]^NT^[S2]^CT^). In the second construct, Gln62 in the Siah1 SBD was mutated to the corresponding Ala62 ([S1-Q62A]^NT^[S2]^CT^). Only the chimera with the P21H/A26P mutations regained partial binding with PHD3. In contrast, the Q62A mutation did not increase PHD3 binding (Fig. 6b), indicating that Ala62 is not critical for the Siah2-PHD3 interaction. Thus, of the 6 dissimilar amino acids in the N-terminal region of the SBD of Siah2 (1–100 aa), we found that His21 or Pro26, or both, play a role in binding to PHD3.

**Figure 7:**
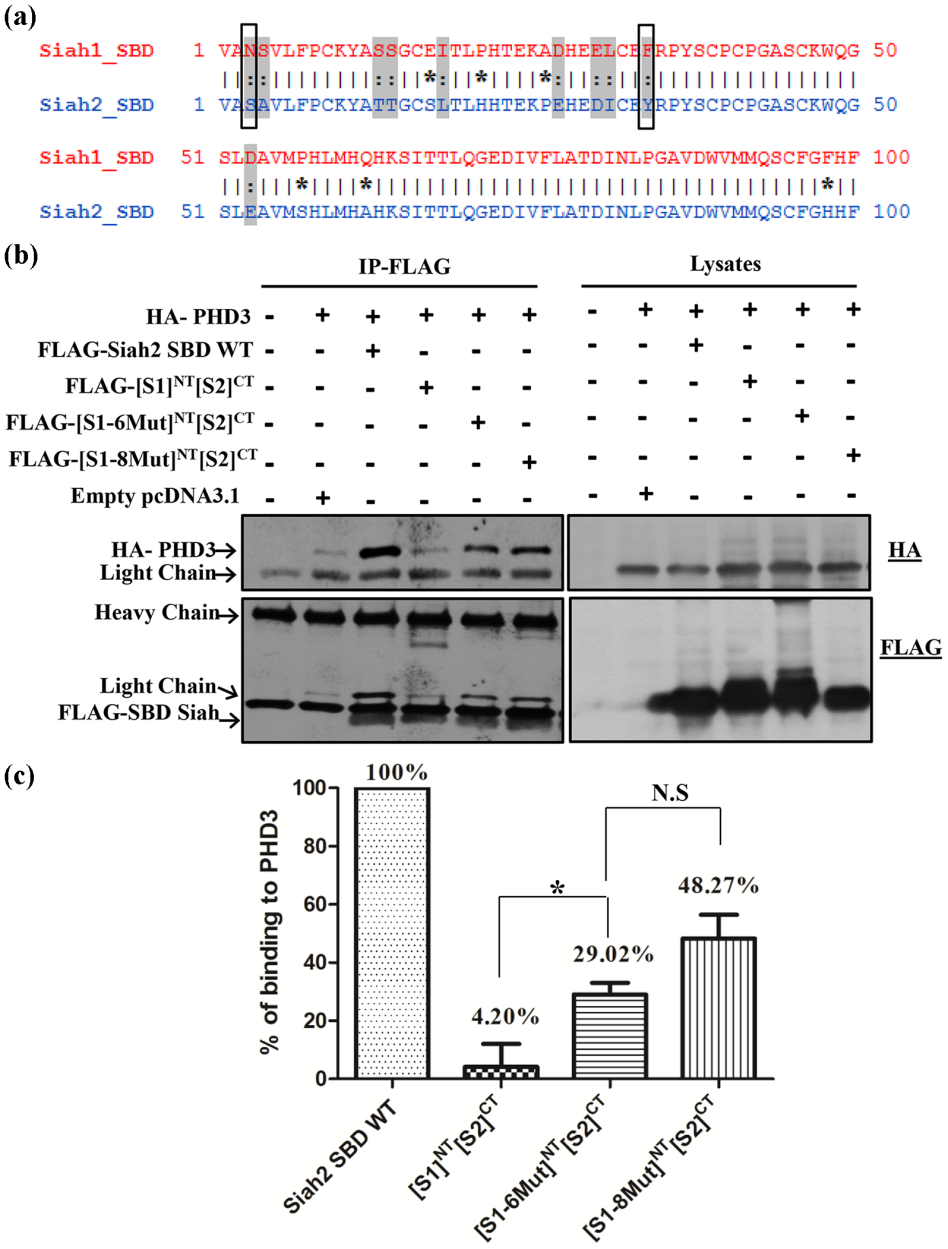
Effect of additional mutations in the N-terminal region of the SBD on binding with PHD3. (**a**) The 10 similar amino acids in the N terminal region (1–00) of Siah1 and Siah2 SBD are highlighted in grey. Mutated residues among the similar amino acids are highlighted within the box. (**b**) HEK293T cells were transfected with the expression plasmids for the indicated proteins. The cells were lysed and the cell lysates were subjected to FLAG-immunoprecipiation (IP). Immunoprecipitates and lysates were then analyzed by western blotting using the indicated antibodies. The [S1-8Mut]^NT^[S2]^CT^ mutant increased binding compared to [S1-6Mut]^NT^[S2]^CT^. (**c**) The amount of PHD3 that coimmunoprecipitated with chimeric and mutated Siah1 and Siah2 SBD was quantified using Gel-pro analyzer software. The binding of the chimeric and mutated SBD to PHD3 was expressed as percentage of the binding of WT Siah2 SBD to PHD3. The data are represented as mean±S.E.M from three independent experiments. Differences in measured variables were assessed with Student's t test. * denotes p<0.05.

Our results also suggest that in addition to His21 and Pro26, other Siah2 residues are important for mediating substrate specificity between Siah1 and Siah2. Out of the 10 similar amino acids in the N-terminal region of Siah1 and Siah2 SBD, we hypothesized a critical role for Ser3 and Tyr34 through their plausible involvement in hydrogen bonding (Fig. 7a). We therefore used the chimera in which all six dissimilar amino acids in the N-terminal region of [S1]^NT^[S2]^CT^ were mutated from Siah1 back to Siah2. In to this [S1-6Mut]^NT^[S2]^CT^ construct, two additional mutations (N3S/F34Y), corresponding to the candidate similar amino acids, were introduced, resulting in the mutant, [S1-8Mut]^NT^[S2]^CT^ (where 8Mut  =  N3S/E17S/P21H/A26P/F34YP57S/Q62A/F98H). Indeed, the [S1-8Mut]^NT^[S2]^CT^ mutant increased binding compared to [S1-6Mut]^NT^[S2]^CT^ (Fig. 7b). Densitometry quantification of the binding affinities revealed that the mutation of the 6 dis similar amino acid increased the binding of [S1]^NT^[S2]^CT^ from 4 to 29 percent. The additional mutation of the two similar amino acids (N3S/F34Y) resulted in a further increase in the binding affinity to 48 percent (Fig. 7c). Taken together, our study suggests that a critical role for the four amino acids Ser3, His21, Pro26, Tyr34, in the N-terminal region of Siah2 SBD in conferring substrate specificity. However, given that introducing these four amino acids into Siah1 results only in a partial recovery of binding, other amino acids are likely to be involved in the interaction between the N-terminal region of Siah2 SBD and PHD3 (see discussion).

## Discussion

The present work attempts to identify the critical residues of Siah2 SBD that determine the preference of PHD3 binding to Siah1 over Siah2. Our results highlight that both the N- and C-terminal regions of the Siah2 SBD are involved in the interaction with PHD3. In the C-terminal region of the Siah2 SBD, Leu121 is critical for selective binding to PHD3. Thus, mutating Glu121, the corresponding residue in Siah1, to Leu121 markedly increases PHD3 binding. In Siah2, the amino acids around Leu121 are hydrophobic and hence we hypothesize that this region might form a hydrophobic pocket or interaction surface that would favour the binding of PHD3. In contrast, in Siah1 this hydrophobic pocket or interphase might be disrupted by Glu121.

**Figure 8:**
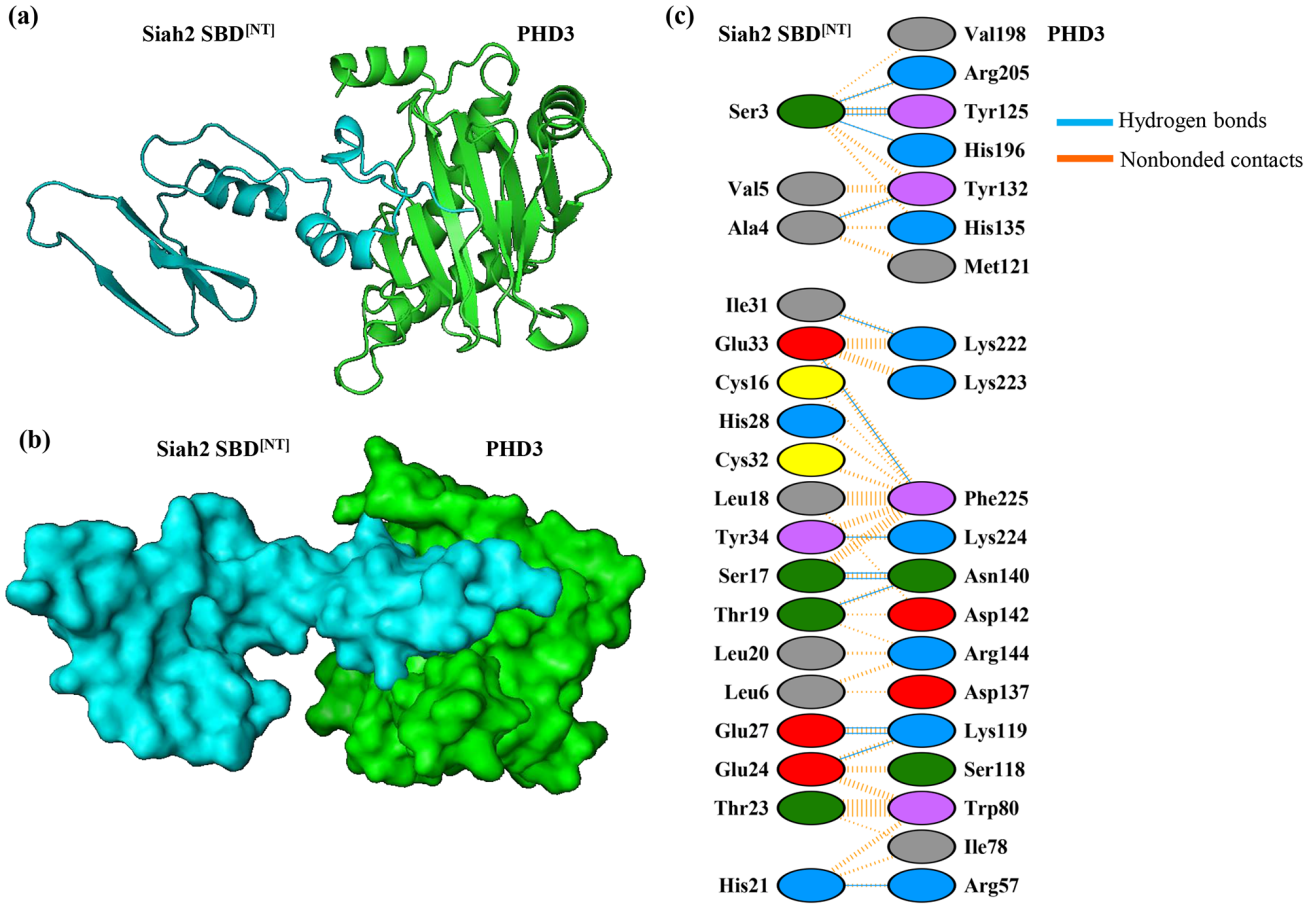
Docking model of the N-terminal Siah2 SBD and PHD3. N-terminal region (1–100) of the modeled Siah2 SBD was docked with PHD3 using an automated Cluspro server. The complex was then presented using Pymol as (**a**) cartoon representation, and (**b**) space filling representation. (**c**) The details of the interactions were obtained by PDBsum. The number of H-bond lines between any two residues indicates the number of potential hydrogen bonds between them. For non-bonded contacts, the width of the striped line is proportional to the number of atomic contacts.

In the N-terminal region of the Siah2 SBD, we could identify four residues (Ser3, His21, Pro26, Tyr34) that are likely to be involved in the binding to PHD3. Substituting the corresponding Siah1 residues with these four amino acids increases PHD3 binding to 48% percent compared to wild type Siah2. In order to identify additional residues that mediate substrate specificity of Siah2 and would restore the binding of Siah1 to PHD3 to 100%, we performed docking studies between the N-terminal region of the modeled Siah2 SBD and PHD3 to obtain a structural perspective. The complex was then analyzed for its detailed interactions using PDBsum. 18 residues of the N-terminal Siah2 SBD and 20 residues of PHD3 were found to be involved in interactions. These include 16 hydrogen bonds and 161 non-bonded contacts (Fig. 8). The 18 amino acids of Siah2 are found to be within the first 35 residues of the N-terminal SBD region. When analyzing the N-terminal sequences of both Siah1 and Siah2 SBD, most of the non-identical amino acids are also present within the first 35 residues of Siah2 SBD. In the docking model, out of the four residues (His21, Pro26, Ser3, Tyr34) that are likely to be involved in PHD3 binding, as determined experimentally, three residues (His21, Ser3, Tyr34) were involved in Hydrogen bonding with PHD3. As proline residues confer conformational rigidity and act as a structural disruptors [43], we hypothesize that Pro26 might cause some structural change in the Siah2 SBD that favours the selective binding to PHD3. Based on the docking analyses shown in Fig. 8c, additional residues in the N-terminal region of Siah2 SBD could be tested in future mutation studies. The corresponding original residue numbers of the reported residues (Leu 121, Ser3, His21, Pro26, Tyr34) are Leu250, Ser132, His150, Pro155, Tyr163 (Fig. 4a).

Evidence from *in vitro*, *in vivo*, and patient sample studies describe opposite roles for Siah1 and Siah2 in cancer progression, metastasis, and therapeutic responses [20]. The different roles of Siah1 and Siah2 are highly likely to be due to the ubiquitination of distinct sets of substrate proteins. Our study helps in understanding the molecular basis of substrate specificity between Siah1 and Siah2 by identifying specific Siah2 SBD residues conferring substrate specificity. This information may be of importance to identify potential inhibitors targeting specifically Siah2, but not Siah1, for therapeutic purposes. It has been reported that targeting the SBD or RING domain using a peptide inhibitor or mutants is capable of reducing tumor growth and metastasis in various models [13], [30], [44]. However, targeting the RING domain is challenging because of the similarity of this domain among the Ring-type E3 ligase family [45] and thus most of the inhibitors for E3 ligases obtained so far inhibit multiple ligases [46]. So far, menadione is the only available Siah2 selective inhibitor, identified from a Meso-scale-based assay of 2000 compounds [44]. However, menadione has multiple other biological activities. The Siah proteins are unique in their SBD architecture, compared to other ligases [47]. Thus, differences in the SBD between Siah1 and Siah2, as highlighted in this study, could be exploited in the future to identify Siah2 specific inhibitors.
